# Exploring the inner roots of discharge against medical advice (DAMA) in an academic psychiatric hospital: a qualitative study

**DOI:** 10.1186/s43045-022-00237-6

**Published:** 2022-09-15

**Authors:** Fatemeh Alidoust, Seyyed Taha Yahyavi

**Affiliations:** Department of Psychiatry, Roozbeh Hospital, School of Medicine, Tehran University Of Medical Sciences, Tehran, Iran

**Keywords:** Discharge against medical advice, Psychiatry, Mental illness, Psychiatric hospital, Qualitative

## Abstract

**Background:**

To explore the roots of DAMA in the Roozbeh Hospital, an academic mental hospital located in Tehran, Iran, the authors conducted a simple qualitative study. Twenty-four in-depth semi-structured interviews with families and eight with patients who were discharged against medical advice were done about the inner motivation and roots of DAMA. The sampling was convenient in trying to reach the maximum variation. The transcribed verbatim of the interviews was coded and categorized inductively by discussion. Ethical consideration was approved by the TUMS ethical committee.

**Results:**

Findings were classified into six categories: The patient’s insistence, miss the beloved one, sense of improvement, outside hospital concerns, dissatisfaction with the quality of medical care, and worries about the hospitalization consequences.

**Conclusions:**

Addressing the inner roots of DAMA can be an opportunity to understand better patients and their families’/relatives’ experiences and feelings. This will give a deep perspective and insight into the subject.

## Background

Discharge against medical advice (DAMA) is the condition that the patients (or in some situations their caretakers/families/relatives) intend to discharge the patients early, despite the physician’s opinion [1]. This phenomenon might be a sign of patients’ dissatisfaction with hospital care or the treatment process [2]. DAMA accounts for approximately 0.1% of post-partum cases to 25% of infectious disease [2]. It is estimated that psychiatric wards are among the highest rates of DAMA. In a recent study, the rate of 20% in India was reported [3], and to the best of our knowledge, the highest rate in literature is 35% [4].

The possible associated factor of DAMA has been mentioned in previous research, which includes hospital service dissatisfaction, financial problems, family problems, caregiver-patient communication, belief in traditional medicine, discomfort with an extended stay in the hospital, and feeling of recovery [5, 6]. According to the fact, the environmental factors play a major role in DAMA, and the generalization of the associated factor in one center should be done with caution [7].

There are pieces of evidence that patients with DAMA are at higher risk for relapse, readmission, complications, and death [6–8]. Therefore, DAMA might increase the total cost of treatment by up to 50% compared to routine discharge [2]. DAMA has some specific characteristics in mentally ill patients. Hospitalized psychiatric patients are usually affected by mood, psychotic, or cognitive symptoms and may not have the capacity for decision-making in critical moments. Under Iranian law, the care and guardianship of patients with severe mental illness (SMI) including schizophrenia, bipolar disorder, and schizoaffective disorders are assigned to their relatives/families that are usually one of their families/relatives. Since most patients admitted to psychiatric hospitals are SMI, as a general regulation, the responsibility for DAMA for all patients admitted to psychiatric hospitals in Iran is assigned to patients’ families/relatives, not to themselves [5]. Therefore, it is logical that in a study that intended to explore the inner roots of DAMA, most of the focus will be on the families/relatives of the patients who are the major agent in DAMA according to rules and regulations.

The present study aimed to explore the inner roots of DAMA in Roozbeh Psychiatric Hospital by using qualitative methods. In the current research, it was sought to focus on the inner feelings and hidden unexplained reasons. An in-depth study of this issue can provide a more accurate image of DAMA roots and might help to a better understanding of the psychiatric patients’ experience of hospitalization.

## Methods

### Setting

Roozbeh Hospital is the oldest academic psychiatric hospital in Iran, located in downtown Tehran. The hospital has 207 active beds in 11 wards and about 3500 admissions annually. This hospital belongs to the Tehran University of Medical Sciences and is a place for training the residents of psychiatry and other medical learners at different levels. About 40 faculty members, 80 residents of psychiatry, and 450 staff are practicing there. Most admitted patients are SMI in relapse or crisis, and the average hospital stay is about 22 days. More than 90% of hospitalizations are via the emergency department and are performed as involuntary hospitalization by the family, court, or Emergency Medical Services (EMS). A quarter of admitted patients withdraw their consent for hospitalization and discharge against medical advice (DAMA).

It is worth noting that the last few months of this study coincided with the COVID-19 pandemic, so some of the content may be directly or indirectly influenced by pandemics. However, it should be emphasized that most of this study was performed before the onset of the pandemic.

### Research design and population

According to the current Iranian laws and regulations, psychiatric patients can only be discharged against medical advice from mental hospitals only by their relatives’/families’ request and they are not allowed to discharge by themselves. Therefore, this is the relatives/families that make decisions for patients’ DAMA. Thus, first, face-to-face, semi-structured, in-depth interviews were conducted with the families/relatives of patients with DAMA. Progressing the interviews, researchers came to the conclusion that according to the obtained codes, it is necessary to interview some of the patients who were discharged against medical advice by their families/relatives. Accordingly, additional interviews were conducted with the DAMA patients themselves. Totally, 24 relatives/families of patients who refer to the hospital for discharging their patients and eight hospitalized patients were interviewed. Most of the participants did not have any academic education. The sampling method was convenient in trying to reach maximum variation. The interviews continued until reaching saturation. The characteristics of participants were displayed in Table 1.

**Table 1:** Characteristics of participants

Number	Relationship with the patients’	Gender	Age	Education
1	Wife	Female	40	HS
2	Father	Male	51	HS
3	Father	Male	56	PS
4	Father	Male	42	HS
5	Father	Male	57	BS
6	Mother	Female	63	PS
7	Mother	Female	48	BS
8	Father	Male	45	HS
9	Brother	Male	40	BS
10	Mother	Female	35	AS
11	Father	Male	57	HS
12	Mother	Female	42	HS
13	Mother	Female	58	PS
14	Mother	Female	54	PS
15	Mother	Female	52	PS
16	Sister	Female	29	BS
17	Sister	Female	39	HS
18	Brother	Male	44	MS
19	Mother	Female	57	PS
20	Grandmother	Female	71	HS
21	Mother	Female	47	BS
22	Sister	Female	40	HS
23	Father	Male	48	BS
24	Husband	Male	60	HS
25	Patient herself	Female	36	HS
26	Patient herself	Female	40	Ph.D.
27	Patient himself	Male	35	PS
28	Patient himself	Male	27	PS
29	Patient herself	Female	30	HS
30	Patient herself	Female	37	PS
31	Patient herself	Female	18	HS
32	Patient herself	Female	57	BS

*PS* Primary school, *HS* High school, *AS* Associate degree, *BS* Bachelor of sciences, *MS* Master of sciences, *Ph.D.* doctor of philosophy

### Data collection

Demographic variables were obtained during interviews. In-depth semi-structured interviews were conducted by a trained senior resident of psychiatry. The interviews were based on the topic guide and covered fields that were mentioned in previous studies as effective factors in patients’ dissatisfaction, such as the quality of therapy and hospital facilities. The interview began with open-ended questions, with clarifications based on patients’ responses, and in-depth content was probed.

### Data analysis

Rigor of the study was increased by checking the content of the interview with participants during the interview. Interviews were transcribed verbatim. Content analysis was inductive. Therefore, the researchers read the transcribed text several times and determined the codes and the content with the agreement, and then the codes were classified, and the main categories were extracted. The analysis was performed using MAXQDA software.

### Ethical issues

The present research was approved by the ethics committee of the medical school of Tehran University of Medical Sciences with the code of ethics: IR.TUMS.MEDICINE.REC.1399.1123. The informed consent was obtained from all interviewees, both for recording the interviews and using the content of the interviews in the research.

## Results

Findings were classified into six categories including the patient’s insistence, miss the beloved one, sense of improvement, financial and family concerns, dissatisfaction with the quality of medical care, and worries about the hospitalization consequences.

### Patient’s insistence

“Patient’s insistence” was the most frequent content given by patients’ families/relatives for DAMA. Some families knew their patients’ reasons for insisting, but many did not. Therefore, researchers found it necessary to interview the patients who insisted on discharge. The contents of this category were derived from a combination of contents, given by both families/relatives and patients themselves. But other categories were merely extracted from families/relatives’ statements.

Major contents of this category include “Fear of being injured by other patients,” “A sense of being fully cured,” “A feeling of being stuck,” “A feeling of monotony,” and Discontent of care.” In explaining these issues, it should be borne in mind that psychiatric patients may feel prone to harm under the influence of symptoms like delusions or hallucinations. Moreover, some inpatients may have unusual or aggressive behaviors which may cause a feeling of fear in other patients. The content of “fear of being injured by other patients” may root in these facts.

Some patients after a few days of being away from the stressful environment and improving their vegetative symptoms like insomnia or anorexia will feel well. They might think that continuing the hospitalization is not necessary. However, doctors may prefer to make the hospitalization longer due to subsiding the signs and improving insights. This may explain the content of “A sense of being fully cured.”

Closed wards, old buildings, and facilities of Roozbeh Hospital in addition to strict regulation of wards may cause “A feeling of being stuck in a tight space” and “A feeling of monotony.”

Since psychiatric medications work gradually and last for several days and non-pharmacological treatments at Roozbeh Hospital are associated with limited facilities, patients may feel that they are not receiving enough care and not much is being done for them. This may be the root of the content of “Discontent of care.”

Some statements by patients or their families are as follows:

- **P1**: He insisted to be out. We did not want DAMA at all. We had brought him to be treated, but he was constantly calling and telling us: take me out, I would not be changed in hospital. I'm not crazy ... He threatened me one day and said: you did not rescue me, I am bored and got nervous and could not endure this situation. I said to myself: "Let him go, let him be out and do whatever he wants", but I promised myself not to live with him anymore."
- **P25**: I think that there was no need for hospitalization at all because I have been arguing with my sister since we were children, and I was just angry that day because of her. I thought I would have a simple routine visit by a doctor. I was completely fine. A physician talked with me and admitted me. If I knew I would be hospitalized, I did not go to the hospital at all, or my mother did not let me. I do not have any certain disease. I just was angry"

### Miss the beloved one

According to limited facilities and resources, visiting the patients by their relatives in Roozbeh Hospital always have been confined to special times and situations. Moreover, due to the COVID-19 pandemic, face-to-face visits became further limited. These conditions made families/relatives and patients miss each other, and experiencing guilt feeling for hospitalization was the root of some DAMA. Some examples of statements made by the participants are as follows:

- P4: "COVID-19 is bothering everyone. For example, if you could visit your son every day and spend hours with him, it would be a better process. Be fair! I really had bad feelings not to visit him and I was more bothered than my son".
- P19: "It's a motherly feeling. She cries a lot and says "take me out, I'm upset, I have a stomachache. I cannot eat well". I decided to take her out this time for showing my kindness. It is a motherly feeling. She has been hospitalized here several times, and doctors eventually discharged her even if I disagreed. Therefore, I prefer to prioritize motherly feelings and emotions this time."

### Sense of improvement

This category explains that the patient’s families/relatives may feel that the patient is relatively well and can be controlled at home, but the physician has not yet reached the conclusion for discharge. Caregivers often feel that they are able to manage the patient at home with a relative improvement in symptoms. They believe they know their loved ones better than the doctors. Sometimes they do not expect much from the patient, and as soon as the acute symptoms such as aggression decrease, it is enough for them, and want the patient to be discharged. Sometimes it is difficult to find an agreement on discharge time. Here, there are some examples of statements:

- P2: “I think he is better. I have to take him home and assess how things are going ... now he recognizes good and bad and is not aggressive, now it is the time to go home I think."
- P17: “I think she is fine, she is not that ill, she was confused and forgot the pills. We decided to hospitalize her and now she is fine. That night he was very ill when we brought her but now, she is ok. "

### Financial and family concerns

This category refers to the issues that occur outside the hospital and may be the reason for DAMA. In recent years, poverty has increased due to the financial crises in Iran, the devaluation of the national currency, and inflation. In this regard, it should be noted that some mentally ill people help provide for the family, which is cut off when they are hospitalized. Moreover, hospitalization itself is costly. Therefore, some families discharge the patients due to financial conditions. Some patients have a relative or a baby outside the hospital that needs their care. Hence, they want to be discharged early. Some examples of interviewees’ statements are as follows:

- P3: "I gave my consent because his insurance booklet was not ready. I cannot pay without insurance."
- P15: "Both the child and she were impatient. I saw that she became better and her child was impatient, and she was homesick, so I did it for her".

### Dissatisfaction with the quality of medical care

This category refers to the fact that sometimes patients feel they were not well cared for. It may be related to an adverse event in the hospital or medication side effects. Furthermore, inappropriate communication can produce these feelings. This inappropriate communication may be due to a lack of communication skills such as active listening and empathy in the staff, or it may be rare due to the patient’s paranoid symptoms. Feelings of not being well cared for may be passed on to the patient’s families/relatives who are usually more concerned about care than the patients themselves.

The following statement is related to the mother of a lady whose daughter had superficially self-harmed in the hospital. This has happened despite routine nursing care.

P12: I brought my daughter to the hospital so that she could not conduct self-injurious behavior. If she was going to self-harm, our home would be a better place! I came to see her and I see that his hands and face are bloody. I was not given a convincing explanation

### Worries about the hospitalization consequences

Some families/relatives are worried that the hospitalization of their patients will have consequences for them. They worry that their relationship with the patient will be damaged after discharge because the patient considers them responsible for involuntary hospitalization. They worry that their patient’s hospitalization in a psychiatric hospital will tarnish the family or the patient’s reputation in the community, making it difficult for them to find a job or a spouse. Some statements are as follows:

- P18: “If the news of my brother's hospitalization in a mental hospital spreads, it does not matter to anyone what was the reason. Our reputation will be ruined. And most of all, it's bad for my sister, who is not married yet. She may miss the good options for marriage...
- P7: "I felt that he had a bad attitude towards me. ‘You are cruel. You are bothering me. I will get worse if I stay here,’ he kept saying yesterday. I'm afraid our relationship will be ruined. "

## Discussion

In the current study, six themes were explored via an interview with psychiatric patients and their families/relatives about the inner roots of DAMA.

One of the expressed categories was the “sense of improvement.” The essence of this concept is that some families/relatives feel improvement earlier than doctors. There are two possibilities here. First, the families/relatives are ready for early discharge, and the physician is conservative and emphasizes the complete remission of signs. It must be considered that the average length of psychiatric hospitalization in Roozbeh Hospital is about 1 month excluding the emergency department and short-staying wards. While most serious signs usually subside within the first few days of hospitalization, this is to be expected that this extended period will exhaust patients and their families/relatives. It is hypothesized that some psychiatrists use defensive psychiatry to avoid malpractice liability. One manifestation of this condition may be delayed discharge of patients and emphasis on the complete improvement of symptoms [9]. If the physician accepts the readiness of patients and discharges them earlier, there may be no need for DAMA [10]. Secondly, it is possible that the information of patients’ families/relatives about the psychiatric disorder and the process of healing is inadequate or they may even deny the problems substantially, and as some of the symptoms have been relieved, they rush to discharge their patient. This concept was replicated in other research [3, 5].

A recent descriptive cross-sectional study conducted at an academic mental hospital investigates the reasons for DAMA. The discussion about this study is important here because the “IRAN psychiatric hospital” is the most similar hospital to Roozbeh Hospital in terms of academics, the culture of patients, location, and administration. The main method of the researchers was questions and answers based on a checklist. They classified the reasons stated by the patients and caretakers into three main factors: (1) patient-related, (2) hospital environment-related, and (3) health care providers related. Some of their findings have been almost replicated in our study including “inadequate nursing care,” “dissatisfaction with medical care,” and “improper communication.” However, according to methodological differences, deep feelings like missing the beloved one, worries, and concerns were not explored [5].

One of the important contents was categorized as “Financial and family concerns”. Financial concerns may relate to insurance coverage and the global financial status of people. In Iran, the majority of people have basic insurance. However, a minority does not. It is postulated that improving insurance coverage can increase compliance and will lower DAMA [11, 12]. In Iran, however, 10% of the cost of treatment is “out of pocket.” On the other hand, in recent years, Iran’s economic crisis has deepened [13, 14]. It is assumed that some patients will not be able to pay for the “out of pocket” section, and currently, charities have limited power and influence [15]. Moreover, when economic conditions are unfavorable, the work of psychiatric patients may be very important to the family and they may want to be discharged the patient early for work. In the current situation, the short-term solution to this issue is to activate as many charities as possible [15].

One of the explored content was dissatisfaction with the quality of medical care. This content is one of the most common and logical reasons for DAMA that have been mentioned in different fields of medicine [16, 17]. The issue of dissatisfaction with the quality of treatment is not limited to DAMA. Most people who are not satisfied with care endure hospitalization until the end of the treatment. In fact, dissatisfaction does not always lead to DAMA [18, 19]. The issue of satisfaction with the quality of care is one of the patients’ rights that should be addressed in detailed research.

One of the expressed content was “worries about the hospitalization consequences.” These consequences refer to the stigma of mental illness and the change in people’s view of the patient and his family, as well as the possibility of the patient’s anger towards the family as a result of hospitalization and the possibility of revenge. In fact, hospitalization may have invisible costs for the family that can affect their lives. This content has not been emphasized in similar studies to the best of our knowledge.

Traditionally, DAMA is postulated as an undesirable event because it has been reported that increases morbidity, readmission, and mortality on the one hand, and on the other hand may cause feelings of failure and challenge in physicians. However, in a research, the value of these assumptions has been questioned. In this study, the idea of patients and their relatives and also health care practitioners about DAMD has been explored. The authors suggested that DAMA could be an opportunity for empathy, empowerment, and care. They suggested that given the patient’s autonomy and the patient-centered medicine, it is better to change the attitudes of healthcare practitioners about DAMA to understand this event as an opportunity for care and empathy, and not to persuade patients or their relatives—even unintentionally—to stay at the hospital [10].

It is difficult to have in-depth interviews with psychiatric patients or their families/relatives who leave the hospital because there is often some dissatisfaction that reduces their cooperation. This probably describes why most existing studies on the subject of DAMA in psychiatric wards have used checklists or closed questions [3–5, 20, 21]. Despite the limitations of qualitative research, asking open-ended questions and addressing the feelings of patients and their families/relatives often gives insights that are not available in other methods. Researchers did not consider “psychiatric diagnosis or symptoms” in exploring the issue so that the interviewer could better address the “experience” regardless of medical terms.

## Conclusions

It should be noted that DAMA often contains feelings and emotional layers. Therefore, addressing these inner roots of this choice can be an opportunity to validate feelings and empathy on the one hand and a better understanding of the patients and their families/relatives’ experiences on the other hand, which together are considered to be an opportunity to improve the doctor-patient relationship. Further exploration of this issue is recommended in future studies.

## Data Availability

Data is available on demand.

## Abbreviation

DAMA: Discharge against medical advice
